# The Role of Portable Air Purifiers and Effective Ventilation in Improving Indoor Air Quality in University Classrooms

**DOI:** 10.3390/ijerph192114558

**Published:** 2022-11-06

**Authors:** Mohammad Aldekheel, Abdulmalik Altuwayjiri, Ramin Tohidi, Vahid Jalali Farahani, Constantinos Sioutas

**Affiliations:** 1 Department of Civil and Environmental Engineering, University of Southern California, Los Angeles, CA 90089, USA; 2 Department of Civil Engineering, Kuwait University, P.O. Box 5969, Kuwait City 13060, Kuwait; 3 Department of Civil and Environmental Engineering, College of Engineering, Majmaah University, Al-Majmaah 11952, Saudi Arabia

**Keywords:** air pollution, indoor air quality, particulate matter (PM), ventilation, ultrafine particles, portable air purifier

## Abstract

In this study we investigated the effectiveness of air purifiers and in-line filters in ventilation systems working simultaneously inside various classrooms at the University of Southern California (USC) main campus. We conducted real-time measurements of particle mass (PM), particle number (PN), and carbon dioxide (CO_2_) concentrations in nine classrooms from September 2021 to January 2022. The measurement campaign was carried out with different configurations of the purifier (i.e., different flow rates) while the ventilation system was continuously working. Our results showed that the ventilation systems in the classrooms were adequate in providing sufficient outdoor air to dilute indoor CO_2_ concentrations due to the high air exchange rates (2.63–8.63 h^−1^). The particle penetration coefficients (P) of the investigated classrooms were very low for PM (<0.2) and PN (<0.1), with the exception of one classroom, corroborating the effectiveness of in-line filters in the ventilation systems. Additionally, the results showed that the efficiency of the air purifier exceeded 95% in capturing ultrafine and coarse particles and ranged between 82–88% for particles in the accumulation range (0.3–2 µm). The findings of this study underline the effectiveness of air purifiers and ventilation systems equipped with efficient in-line filters in substantially reducing indoor air pollution.

## 1. Introduction

Improving indoor air quality using air filtration technologies is essential since people spend most of their time in closed environments [1,2,3,4]. Indoor air pollution leads to adverse health outcomes and almost 3.8 million premature deaths annually [5]. Occupants’ exposure to indoor particle pollutants can cause a number of adverse health effects, including respiratory illnesses, lung cancer, strokes, heart failure, asthma, and eye problems [6,7,8,9,10]. In addition to the health drawbacks, indoor pollution in working environments can lead to fatigue and a decline in focus and productivity [11].

In addition to ambient particles infiltrating indoors and particles emitted by indoor sources, human-generated particles (i.e., airborne aerosol particles released by the exhaled breath of humans) are major routes of airborne transmission of bacteria and viruses, including SARS-CoV-2, especially in confined environments with high population density, such as classrooms [12,13,14,15]. The exhaled particles generally have an aerodynamic diameter of less than 1 μm, mostly in the range of 0.1 to 0.5 μm [16,17,18]. The larger exhaled droplets settle on the ground within seconds due to the gravitational force and/or evaporate to smaller particles in a few seconds [19]. The smaller particles remain suspended in the indoor environment for several hours and can be carried by air currents as far as several meters from their source [15,20]. These smaller particles have a greater capacity to increase the infection potential than large particles since they can travel longer distances [21]. 

Given the health impacts caused by indoor airborne pollutants, employing air purification means in indoor spaces is essential for decreasing pollutant concentrations [15,18]. There are two main methods used to enhance indoor air quality and remove indoor particle pollutants, including in-line filters in ventilation systems and portable air purifiers [22]. Portable air purification units have been widely used in recent years due to their efficient removal of indoor pollutants [23,24,25]. They have been placed in approximately 30% of private residential buildings in developed countries, and a steady growth in the use of these cleaning devices is expected in the upcoming years [1,24]. The existence of in-line filtration in mechanical ventilation systems reduces the infiltration of outdoor particles to indoor spaces to a certain level depending on the filter’s characteristics, filter efficiency, and particle size [26,27,28]. The effectiveness of these in-line filters in capturing ambient particles is assessed by the penetration coefficient (P), which describes the fraction of outdoor particles that successfully penetrate the building into the indoor environment [26,29,30]. Moreover, the adequacy of the ventilation systems in bringing sufficient outdoor air to the indoor environment is assessed by the air exchange rate value [31,32]. Air exchange rate (*AER*) is the rate at which indoor air is entirely replaced by outdoor air in a specific closed environment (e.g., classrooms). The replacement of indoor air with outdoor air occurs by various means, such as natural ventilation (e.g., doors and windows) and forced ventilation (e.g., mechanical ventilation systems). Indoor air quality can be improved by increasing the air exchange rate, since allowing more air to enter the space will dilute indoor pollution, except in cases where outdoor pollution is substantially high [31,33] in which the outdoor air needs to be purified by some sort of in-line filter. 

The main objective of this study was to explore the effectiveness of air purifiers and mechanical ventilation systems equipped with in-line filters in removing indoor airborne particles originating from outdoor and indoor sources in university classrooms. Several studies supported the effective work of the air purifier inside classrooms in improving indoor air quality and mitigating the transmission of bacteria and viruses [34,35,36,37]. However, this study provided additional insights by examining the performance of both air purifiers and in-line filters in the ventilation systems working simultaneously inside various university classrooms with different characteristics. In addition, we investigated the adequacy of the ventilation systems in bringing sufficient outdoor air to the indoor environment. The findings of this work provide significant insights for public health officials, especially in educational institutions, to implement air pollution control systems and enhance the quality of air in indoor environments.

## 2. Methods

### 2.1. Measurement Sites and Protocol 

**Figure 1:**
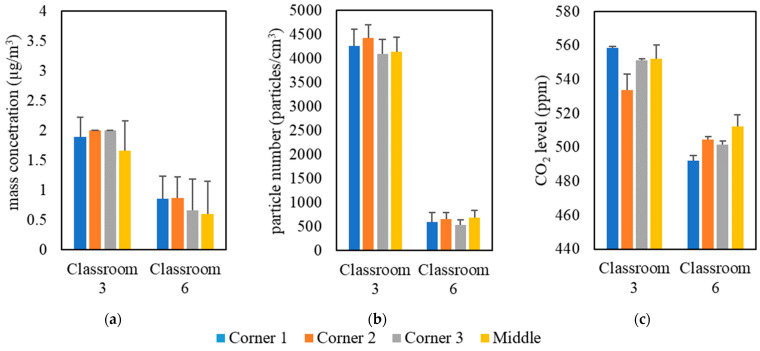
Spatial variability in classrooms 3 and 6 based on (**a**) PM, (**b**) PN, and (**c**) CO_2_. The error bars indicate standard deviations of values measured in a single day.

The measurements were conducted inside classrooms in the University of Southern California (USC) main campus area over a 5-month period from September 2021 to January 2022. Table 1 shows the details of the selected classrooms, including volume, floor area, and the total number of students. These classrooms were solely dependent on forced ventilation (i.e., mechanical ventilation systems) as the means of air exchange between outdoor and indoor environments. Natural ventilation was minimized in all classrooms by closing all doors and windows. The indoor monitoring was performed in two separate campaigns; the first campaign was held in all selected classrooms with students attending classes, while the second campaign was conducted in an empty classroom (i.e., classroom 3). In the first measurement campaign, indoor air quality measurements were conducted in three phases in 9 classrooms located in 7 different buildings; each phase had a different setting of an air purifier (Model Trio Plus^TM^, Field Controls, Kinston, NC, USA) equipped with H13 HEPA filters. The first phase was carried out without the presence of the portable air purifier to evaluate the effectiveness of ventilation systems equipped with in-line filters in reducing indoor pollutant levels without the interference of additional air-cleaning devices. In the second phase, we conducted the measurements while both the classroom’s ventilation system and the air purifier (with flow rate of 267 m^3^/h) were active. In the third phase, the air purifier was set at the highest possible flow rate (i.e., 748 m^3^/h) while the ventilation system was operating simultaneously. We performed real-time measurements for indoor and outdoor PM_2.5_ mass concentrations (PM), particle number concentrations (PN), and CO_2_ levels during active 2-hour lectures in the presence of students. It should be noted that strong indoor particle generation sources (e.g., chalkboard dust and cleaning activities) were not present in the classrooms during the lectures. Pollutants’ monitoring in each classroom started 15 min before the beginning of the lecture and continued until 15 min after the end of the lecture. On the same day, we also monitored the outdoor pollutant concentrations for 15 min before and after the lecture to ensure that the outdoor concentration had not changed considerably while the lecture was ongoing. For each classroom, we repeated the previous procedure on three different days by changing the configuration of the purifier according to the three phases discussed earlier. Moreover, the location of the monitoring devices in the classrooms could affect the readings of the indoor pollutant concentrations. Therefore, we investigated the spatial variance in the pollutant concentrations by placing the monitoring devices in the middle and corners of the classrooms, the results of which are shown in Figure 1 for two different classrooms as an example (the rest of the classrooms yielded similar results). The results indicate overall spatial homogeneity for PM, PN, and CO_2_ concentrations inside the classrooms. This observation shows that the particle and gas pollutants are well mixed due to the overall high air exchange rates in the classrooms; thus, the location of the measuring devices in different spots within the classroom should not result in notable differences between the readings. According to the findings of Küpper et al. (2019) [22] regarding the possible spatial variance in the purifier’s removal efficacy, changing the location of the purifier will provide almost identical removal efficiencies and lead to the same distribution of clean air in the space. Therefore, we positioned the purifier in a fixed location (i.e., the center of the classroom) during the entire campaign.

The second measurement campaign was carried out in classroom 3 in the presence of an indoor pollution source (i.e., sodium chloride aerosols) to simulate exhaled particles of humans. Particles can be generated by humans through various activities, including breathing, speaking, coughing, and sneezing. The particle size that is generally produced by breathing ranges between 0.1 and 1.0 μm [17,38,39,40]. On the other hand, coughing, sneezing, and speaking generate larger particles compared to breathing; these particles are typically larger than 5 μm and will either settle gravitationally or evaporate to smaller particles (<1 μm) [19,34,41,42]. To corroborate the use of sodium chloride (NaCl) as a representative for human exhaled particles, we measured the size distribution of NaCl particles by means of an optical particle sizer (OPS) (Model 3330, TSI, Shoreview, MN, USA) and a scanning mobility particle sizer (SMPS) (Model 3936, TSI, Shoreview, MN, USA). At first, we prepared a suspension by dissolving 50 mg of sodium chloride in 100 mL of ultrapure Milli-Q water to reach a concentration of 500 µg/mL. The suspension was sonicated in an ultrasonic bath for 30 min to achieve a homogenous solution. NaCl suspension was subsequently aerosolized using a commercially available nebulizer (Model 11310 HOPE^TM^ nebulizer, B&B Medical Technologies, Carlsbad, CA, USA) that was connected to a compressor pump (Model VP0625-V1014-P2-0511, Medo Inc., Roselle, IL, USA) equipped with a HEPA capsule (Model 12144, Pall Corporation, USA) to supply compressed filtered air to the nebulizer. The nebulizer was connected to both the SMPS and OPS by a clear vinyl tube to obtain the number-based particle size distribution. The particle size distribution is shown in Figure S1 and indicates that NaCl particles mostly fall in the range of 0.071 to 1.13 µm with a peak at 0.51 µm, which supports the use of NaCl as a representative of the particles generated by humans. A number of previously published studies used NaCl as the aerosol test agent to assess the effectiveness of air filtration means [18,43,44,45,46]. In addition, the National Institute for Occupational Safety and Health (NIOSH) considered NaCl as a standard test aerosol for measuring the effectiveness of respiratory protective equipment (e.g., N95 masks) [47]. Following the same setup and sample preparation discussed earlier, NaCl suspension was aerosolized in classroom 3 to act as an indoor source of aerosols.

### 2.2. Instrumentation 

Various air quality monitors were used in this study to measure different pollutant concentrations. We employed the DiSCmini nanoparticle counter (Model Testo DiSCmini, Testo, West Chester, PA, USA) in our experiments to measure particle number concentrations. The TSI DustTrak monitor (Model 8520, TSI, Shoreview, MN, USA) was used to obtain real-time PM_2.5_ particle mass concentrations. In addition, we employed a Q-track device (Model 8551, TSI, Shoreview, MN, USA) to measure indoor and outdoor CO_2_ levels. One of the objectives of the second measurement campaign in the empty classroom was to estimate the purifier’s efficiency for each particle size. This was done using the optical particle sizer (OPS) (Model 3330, TSI, Shoreview, MN, USA) to obtain particle concentrations and size distributions. The OPS measures particle sizes from 0.3 to 10 μm, which are particles in the coarse and accumulation size ranges. Further details about the calibration of the monitoring instruments are available in the supplementary material.

### 2.3. Additional Calculations

#### 2.3.1. Indoor Particle Penetration (P) in the Set of Classrooms

The indoor particle penetration (P) was calculated based on the steady-state approach, which is similar to that of Chao et al. (2003) [48]. Treating the classroom as a closed system allows for the application of the mass balance equation. Equation (1) illustrates the mass balance applied in the tested classrooms:

(1) $$\frac{dC_{in}}{dt}=\frac{S}{V}+C_{out}AER\;\left(P\right)-C_{in}AER-C_{in}k$$

where *dC_in_*/*dt* is the change of indoor particle concentration with time, *S* represents the indoor particle production rate, *V* is the volume of the classroom (m^3^), *k* is the deposition rate of particles (h^−1^), and *C_in_* and *C_out_* are the indoor and outdoor particle concentrations, respectively. The indoor particle production rate in Equation (1) was neglected (i.e., S = 0) since there was no indoor source for particles in the studied classrooms during the active lectures. The presence of students inside the classrooms did not result in noticeable increases in the indoor particle concentrations since the particle emission rate by humans is negligible compared to the particles infiltrating from outdoor sources [49,50,51]. The indoor particle concentration in the classrooms reached a steady-state condition after 5–8 min from the beginning of the lecture (i.e., *dC_in_*/*dt* = 0), which means Equation (1) can be rearranged to the following equation:

(2) $$P=\frac{C_{in}\left(AER+k\right)}{C_{out}AER}$$

The above expression has been widely used for the calculation of effective penetration or the steady-state indoor concentration (*C_in_*) in numerous previous studies [52,53,54]. The calculation of particle penetration indoors was carried out in the first phase of measurements when the air purifier was switched off. The particle penetration should not be affected by using the air purifier in the second and third phases of measurements. However, to show the agreement of penetration coefficients in the three phases, the following equation was used when the air purifier was active:

(3) $$P=\frac{C_{in}\left(AER+k+\frac{CADR}{V}\right)}{C_{out}\;AER}$$

where *CADR* is the clean air delivery rate of the purifier (m^3^/h), and *V* is the volume of the classroom (m^3^). Although the operation of an air purifier does not affect the penetration coefficient, it significantly affects the indoor-to-outdoor ratio. Equation (3) demonstrates that the addition of the (*CADR*/*V*) term will decrease the (*C_in_*/*C_out_*) ratio. Moreover, increasing the purifier’s flow rate leads to a further reduction in the indoor-to-outdoor ratio.

The penetration coefficient in the studied classrooms was used as a metric for assessing the effectiveness of the in-line filters of the ventilation systems in capturing particles penetrating the building from the outdoor space. The air handling units in all tested classrooms, except classroom 3, were equipped with a dual direction 12-inch MERV 14 filter with a fiberglass media (Model Aerostar FP Mini-Pleat, Filtration Group, Santa Rosa, CA, USA). MERV 14 efficiently filters the outside air and the air returning from the indoor space. The air handling unit of classroom 3 had a 2-inch MERV 13 filter with a synthetic air media (Model Aerostar Green Pleat, Filtration Group, Santa Rosa, CA, USA). According to the manual of Aerostar filters, MERV 13 filters have lower particle removal efficiency than MERV 14 filters. 

#### 2.3.2. Air Purifier’s Efficiency in Classroom 3

The second measurement campaign consisted of three stages leading to the determination of the purifier’s efficiency. In the first stage, the background indoor pollutant concentrations were measured without operating the pollution source and the purifier. The second stage started when the indoor pollutant generator was switched on until a stabilized particle concentration was reached. In the third and last stage, the indoor pollutant source was switched off, and the air purifier was switched on. The purpose of the third stage was to investigate the particle decay rate (K_purifier_) in the presence of the air purifier. The experiment was repeated three times by changing the third-stage scenario. In the first scenario, the purifier was switched off in order to measure the natural decay rate of particles (K_natural_) when the ventilation system was only switched on. In the second scenario, the purifier was operated at a flow rate of 267 m^3^/h to obtain the particle decay rate (K_purifier (low)_). The last scenario was conducted while operating the purifier at a flow rate of 748 m^3^/h to measure the decay rate at the purifier’s maximum fan speed (K_purifier (high)_). In order to calculate the particle decay rate after switching off the NaCl source, we treated the classroom as a closed system and applied the mass balance equation below:

(4) $$\frac{dC_{in}}{dt}=C_{out}AER\;\left(P\right)-C_{in}\left(K\right)$$

where *dC_in_*/*dt* is the change of the indoor particle concentration with time, *K* is the particle decay rate (h^−1^), *AER* is the air exchange rate (h^−1^), *P* is the particle penetration coefficient, and *C_in_* and *C_out_* are the indoor and outdoor particle concentrations, respectively. Based on the integration of Equation (4), the general equation for the indoor concentration is expressed as follows:

(5) $$C_{in\left(t\right)}=\frac{C_{out}AER\;\left(P\right)}{K}\left(1-e^{-\left(K\right)t}\right)+C_{in\left(o\right)}e^{-\left(K\right)t}$$

where *C*_*in*(*t*)_ is the concentration of the particles at time *t* and *C*_*in*(*o*)_ represents the concentration of the particles at time 0. In order to analyze the decay of the particles (i.e., reduction in particle concentration) with time, we subtracted the concentration of the particles continuously infiltrating indoors (i.e., the first term of Equation (5)) from the measured concentrations during the decay period. This allowed us to use the exponential equation below to obtain the decay rate of the particles:

(6) $$C_{in\left(t\right)}=C_{in\left(o\right)}e^{-\left(K\right)\;t}$$

The particle decay rate is a function of the air exchange rate, particle deposition rate, and particle removal rate by the purifier. Thus, Equations (7) and (8) were used to express the decay rate in the natural condition (i.e., without the purifier) and in the presence of the purifier, respectively:

(7) $$K_{Natural\;}=AER+k$$

(8) $$K_{Purifier\;}=AER+k+\eta \frac{Q}{V}$$

where *AER* is the air exchange rate (h^−1^), *k* is the particle deposition rate (h^−1^), *η* represents the purifier efficiency, *Q* is the air volumetric flow rate of the purifier (m^3^/h), and *V* represents the volume of the classroom (m^3^). By combining Equations (7) and (8), we can calculate the purifier’s efficiency, as shown in Equation (9):

(9) $$\eta =\frac{{\left(K_{Purifier\;}-K_{Natural}\right)}_{V}}{Q}$$

The decay in the particle mass and number concentrations was plotted as a function of time after switching off the aerosol source. Decay curves were obtained for a range of particle sizes (0.3–10 µm) to estimate the purifier’s efficiency in removing different particle sizes. In addition, the purifier removal efficiency for ultrafine particles was estimated using PN data obtained from the DiSCmini since it mainly detected particles with diameters below 0.3 µm. 

## 3. Results and Discussion

### 3.1. Indoor Monitoring of PM, PN, and CO_2_ Concentrations in the Set of Classrooms 

#### 3.1.1. Indoor CO_2_ Levels

**Figure 2:**
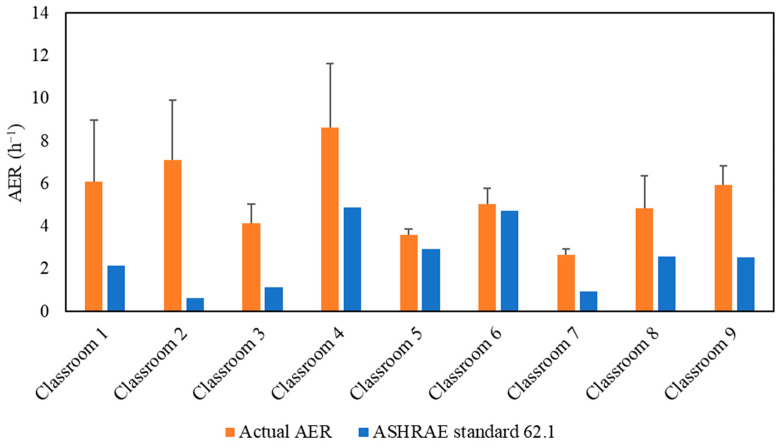
Air exchange rate (*AER*) values for the tested classrooms. The error bars indicate standard deviations of the values measured on three different days.

Figure 2 demonstrates the actual air exchange rates (*AER*) in the selected classrooms, which were measured and showed a very good agreement with the *AER* received from the USC facilities and the management department as shown in Figure S2. The detailed methodology of calculating the *AER* inside the classrooms is described in the supplementary material. *AER* is the metric for assessing the adequacy of the applied ventilation (i.e., mechanical ventilation system) in bringing in sufficient outdoor air and diluting indoor CO_2_ concentrations. However, high air exchange rates will also increase the infiltration of outdoor particulate pollutants, especially if the ventilation system operates without an in-line filtration system that removes a fraction of outdoor particle pollutants [26,31]. As shown in Figure 2, the classrooms’ *AER* values ranged from 2.63 h^−1^ (Classroom 7) to 8.63 h^−1^ (Classroom 4). The American Society of Heating, Refrigerating and Air-Conditioning Engineers (ASHRAE) standard 62.1 (2016) recommended a minimum ventilation rate of 7.5 L/sec (27 m^3^/h) per person in closed environments [55]. Figure 2 shows the alignment of the *AER* values with the ASHRAE’s recommendation in all investigated classrooms. Therefore, these *AER* values indicate sufficient outdoor-to-indoor air circulation and adequate ventilation. 

**Figure 3:**
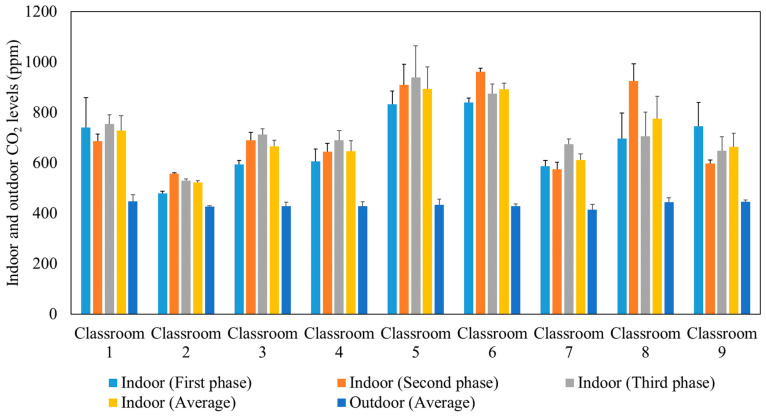
Average outdoor and indoor CO_2_ levels during the three phases in the studied classrooms. The error bars indicate standard deviations of values measured in a single day.

After approximately 10 min from the beginning of each lecture, the indoor CO_2_ concentration reached a well-mixed steady-state condition when the production of CO_2_ by the students was equal to the losses of CO_2_ due to air circulation in the ventilation system. The ASHRAE standard 62.1 (2016) recommended that the indoor steady-state CO_2_ concentration should not exceed the outdoor CO_2_ level by more than 700 ppm [55]. Figure 3 presents the average outdoor and indoor CO_2_ concentrations during the three phases of measurements in the studied classrooms. The comparable indoor CO_2_ levels in the three phases confirm that the indoor CO_2_ concentrations are not affected by the use of air purifiers since the latter remove particulate and not gaseous air pollutants. Elevated concentrations of CO_2_ can impact productivity [56,57,58] and lead to headaches, tiredness [59,60], and sick building syndrome (SBS) symptoms (e.g., difficulty in concentration, dizziness) [61,62,63,64]. According to the recommended indoor CO_2_ level by ASHRAE (not exceeding the outdoor level by 700 ppm) and the measured outdoor CO_2_ level (400–500 ppm), the indoor CO_2_ levels in the tested classrooms should not exceed 1100–1200 ppm. This is consistent with our measurements inside the classrooms which showed values ranging between 500 ppm and 900 ppm. This observation corroborates that the ventilation systems in all the tested classrooms are adequate and provide sufficient outdoor air to dilute indoor CO_2_ concentrations as a result of the generally high air exchange rates (2.63–8.63 h^−1^) in each classroom [32,65,66].

#### 3.1.2. Particle Mass and Number Concentrations and Indoor-to-Outdoor Ratios inside the Classrooms

Table 2 summarizes the ambient, indoor, and indoor-to-outdoor (I/O) ratios of PM_2.5_ mass concentrations and particle number concentrations in the occupied classrooms for the first, second, and third measurement phases. During the first phase, classroom 3 exhibited the highest indoor PM_2.5_ mass concentration (8.62 µg/m^3^), followed by classroom 9 with an indoor mass concentration of 2.43 µg/m^3^. The indoor mass concentration during the three phases does not accurately reflect the effectiveness of the air purification unit in reducing indoor pollution because the ambient pollution has a significant influence on the indoor concentration. For example, classroom 9 showed a higher PM indoor concentration (2.43 µg/m^3^) compared to classroom 8 (0.95 µg/m^3^) in the first phase, while the corresponding outdoor levels were 21.67 and 5.69 µg/m^3^, respectively. Therefore, we used the indoor-to-outdoor ratio as a metric for measuring the effectiveness of ventilation and air purifiers in reducing indoor pollutant levels. Excluding classroom 3, all classrooms had PM and PN I/O ratios below 0.2 in the first phase without using the purification unit. This observation indicates that the ambient PM and PN were initially reduced by 80% or more in most classrooms by just the in-line filters of the ventilation system. In classroom 3, the ambient PM and PN concentrations in the first phase were reduced by 56 % and 65%, respectively. The low I/O values in the first phase did not allow for a proper investigation of the purifier’s efficiency in removing particles in the subsequent phases. For example, the PN I/O ratio in classroom 4 decreased from 0.05 in the first phase to 0.04 in the third phase when the purifier was operated at the maximum volumetric flow rate (748 m^3^/h). Starting with a low I/O value did not allow the purifier to reduce the I/O ratio substantially and, more importantly, the indoor PM levels approached the limit of detection of the DustTrak, such as classrooms 2 and 7.

**Figure 4:**
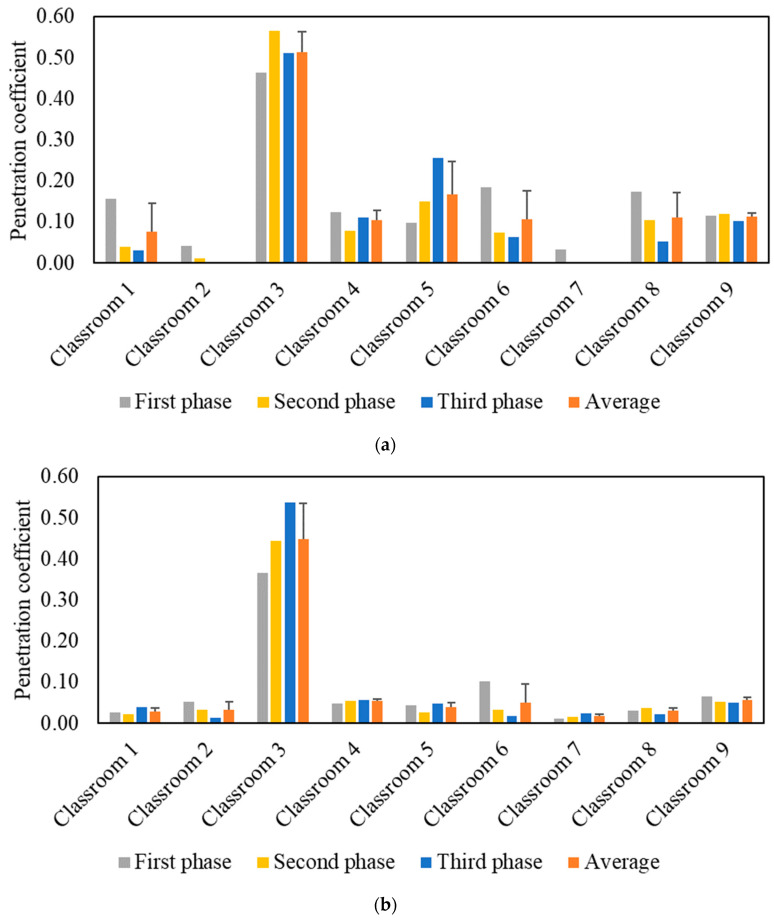
Particle indoor penetration based on: (**a**) PM_2.5_ mass concentration and (**b**) particle number concentration. The error bars indicate standard deviations of the values measured on three different days.

The effective indoor penetration was measured for each classroom to assess the effectiveness of the in-line filtration in the air handling units. Figure 4 shows the penetration coefficients for PM and PN during each phase, as well as the average values throughout all three phases. Unlike the I/O ratio, the penetration coefficient values are independent of the purifier as corroborated by the comparable values in the three phases. The penetration coefficients for PM were higher than PN as the latter primarily consists of ultrafine particles (i.e., size < 0.3 µm), which are easier to remove by filters due to their diffusivity. The P values in the majority of classrooms were low for both PM (<0.2) and PN (<0.1), which can be attributed to the presence of efficient in-line filters (i.e., MERV 14) in the ventilation systems of almost all classrooms. Higher penetration coefficient values for PM (0.51) and PN (0.45) were observed in classroom 3 due to the less efficient in-line filter (i.e., MERV 13) used in its mechanical ventilation system. Based on the penetration values in classroom 3, the in-line filtration system could only reduce ambient PM and PN by approximately 49% and 55%, respectively. Therefore, we selected classroom 3 to conduct our experiments for the second measurement campaign. 

### 3.2. Indoor Monitoring of PM, PN, and CO_2_ Concentrations in Classroom 3 in the Presence of Indoor Particle Pollution Source 

Real-time monitoring of PM, PN, and CO_2_ was conducted in the presence of an aerosol-generating source emitting sodium chloride in classroom 3. As discussed earlier, classroom 3 was selected for the second measurement campaign due to its higher penetration coefficient compared to the other classrooms. The measured indoor CO_2_ level in classroom 3 was constant during the three stages due to the absence of indoor CO_2_ sources (e.g., students). Indoor CO_2_ levels were not affected by the generation of aerosols or the change of the purifier setting, as we would expect; however, PN and PM concentrations were heavily affected. 

#### 3.2.1. PM and PN Decay Rates with and without the Use of Air Purifier at Different Volumetric Flow Rates

**Figure 5:**
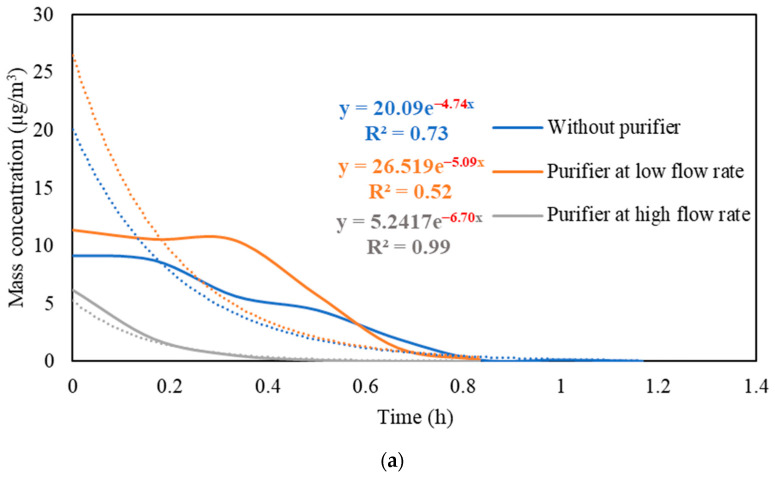

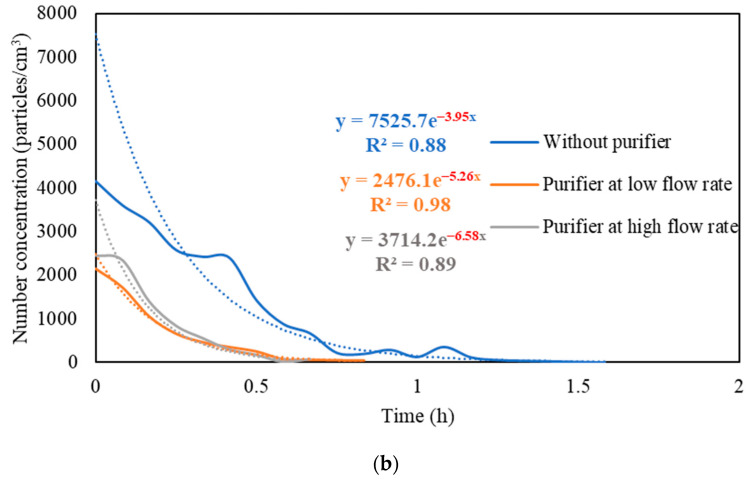
Decay rate curves with and without the use of purifier at different flow rates based on (**a**) PM and (**b**) PN in classroom 3. The dotted curves represent exponential trendlines.

Figure S3 presents the real-time measurements of PM and PN concentrations during the three stages: (i) background condition, (ii) NaCl indoor source is switched on, (iii) purifier is switched on and the source is switched off. It also shows the PM and PN measurements for different configurations of the third stage (i.e., without the purifier, the purifier at a low flow rate of 267 m^3^/hr, and the purifier at a high flow rate of 748 m^3^/h). Figure 5 shows the PM and PN particle decay curves in classroom 3, which were obtained and analyzed based on the third-stage data. The natural decay rates of the particles without the application of the air purifier were in the range of 3.9 to 4.8 h^−1^, where the K values were 4.74 h^−1^ and 3.95 h^−1^ for PM and PN, respectively. When the purifier was switched on at a low flow rate (267 m^3^/h), the decay rates increased to 5.0–5.3 h^−1^, with K values of 5.09 h^−1^ for PM and 5.26 h^−1^ for PN. Operating the purifier at the maximum air flow rate (748 m^3^/h) resulted in a significant increase in the particle decay rates (6.5–6.7 h^−1^), with decay values of 6.70 h^−1^ and 6.58 h^−1^ for PM and PN, respectively. The theoretical values of the decay rates were calculated using Equations (7) and (8). According to Long et al. (2001) [54], the deposition rate is dependent on the particle size and ranges between 0.10–0.25 h^−1^ for PM_2.5_ particles. Table 3 shows a good agreement between the theoretical and experimental decay rates for PM.

The quick reduction in particle concentrations clearly demonstrates the effectiveness of the air purifier. In the first stage, the initial PM and PN concentrations at the beginning of the decay period reached a 50% reduction after 35–40 min when only mechanical ventilation was on. Using the purifier at a low flow rate of 267 m^3^/h and a high flow rate of 748 m^3^/h reduced the particle number concentrations by 50% after 25–30 min and 10–15 min, respectively. According to Szabadi et al. (2022) [18], operating the purifier at the maximum flow rate caused a 50% reduction in the particle number concentration after 20 min of switching off the aerosol source, which is consistent with our study. Lower decay rates will result in longer particle residence times indoors and, if these aerosols contain viruses (e.g., SARS-CoV-2), the probability of transmission and infection will increase [35,36,44,67]. Zuraimi et al. (2011) [44] reported that using an air purifier at its maximum fan setting reduced the residence time of coughing and sneezing particles from 4–6 h to 30–40 min. All the aforementioned studies support the use of an air purifier at the maximum flow rate to increase the particle decay, which will decrease the risk of viruses’ transmission in case an infectious person is present in the classroom. 

#### 3.2.2. Removal Efficiency as a Function of Particle Size

**Figure 6:**
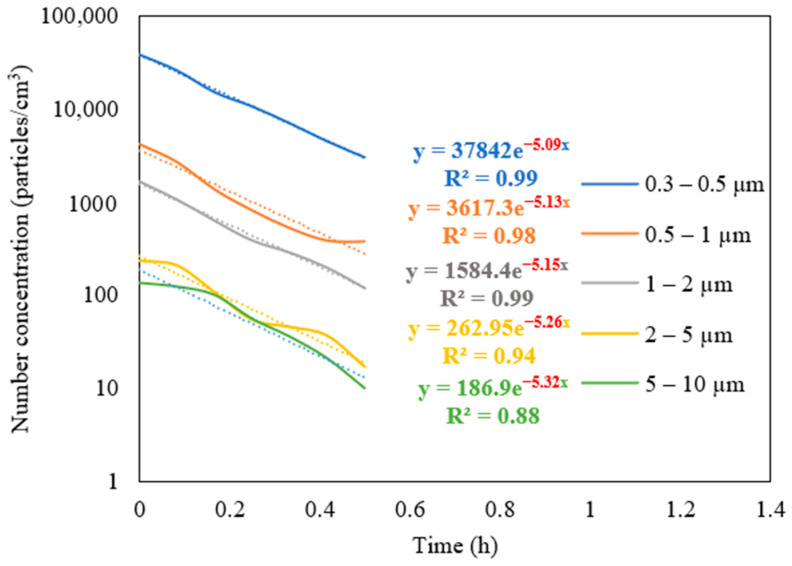
Decay rate curves as a function of particle size in classroom 3 after switching off the NaCl source and operating the purifier at a flow rate of 267 m^3^/h. The dotted curves represent exponential trendlines.

**Figure 7:**
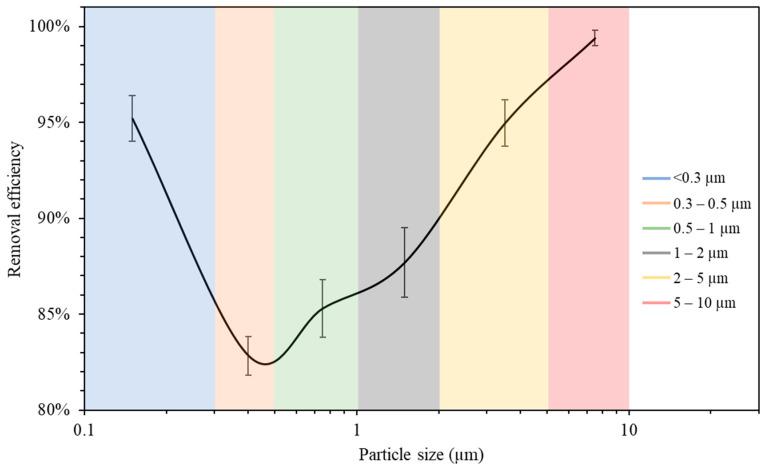
Air purifier removal efficiency as a function of particle size. The error bars indicate standard deviations of values measured in a single day. Values in x-axis are the mid-point diameters of each particle size range.

The measurements of the particle number concentration at the purifier’s flow rate of 267 m^3^/h were used to determine the purifier’s removal efficiency as a function of particle size in classroom 3. Figure 6 presents different particle decay rates based on various particle size ranges. As shown in the figure, the increase in particle size is associated with a higher value of particle decay rate. The efficiencies for each particle size range were calculated and shown in Figure 7. The particle removal efficiencies of the purifier for the size ranges (0.3–0.5 μm), (0.5–1 μm), (1–2 μm), (2–5 μm), and (5–10 μm) were 82.8%, 85.3%, 87.7%, 95.0%, and 99.4%, respectively. Higher efficiencies were achieved for coarse particles, which indicates the efficient performance of HEPA filters in capturing coarse particles. HEPA filters are less efficient in removing particles in the accumulation mode (0.3–2 μm), with removal efficiencies between 82% and 88%. 

The measurements of the particle number concentration in Figure 5b were used to assess the removal efficiency of ultrafine particles since PN data were dominated by particles with a size range of less than 0.3 μm. The decay rate at the purifier’s flow rate of 267 m^3^/h was calculated as 5.26 h^−1^, corresponding to a removal efficiency value of 95.2%. These results lead to the conclusion that the air purifier equipped with HEPA filters is more efficient in removing both ultrafine particles (<0.3 μm) and coarse particles (2–10 μm). However, particles in the intermediate size range (0.3–2 μm) were somewhat less efficiently removed compared to those in the coarse and ultrafine ranges, although the removal efficiency even in that particle range was between 82 and 88%. These results are consistent with various previously published studies and are a result of the fact that smaller particles are easily removed by filters due to their high diffusivity, and larger particles are primarily removed because of their high interception and inertia impaction [68,69]. 

## 4. Summary and Conclusions

This work investigated the effectiveness of air purifiers working in conjunction with in-line filters of mechanical ventilation systems inside different classrooms and their role in improving air quality and capturing pollutants originating from both indoor and outdoor sources. The mechanical ventilation systems in all classrooms, except one, were equipped with 12-inch MERV 14 filters that significantly reduced ambient PM and PN concentrations by more than 80%. The less efficient in-line filter (MERV 13) in the ventilation system of classroom 3 reduced ambient PM and PN by 49% and 55%, respectively. The indoor CO_2_ levels in the analyzed classrooms (500–900 ppm) were below the ASHRAE 62.1 standard, indicating adequate ventilation and sufficient outdoor-to-indoor air circulation due to the high air exchange rates (2.63–8.63 h^−1^). Moreover, operating the purifier at the maximum flow rate (748 m^3^/h) in classroom 3 resulted in increasing the particle decay rate from 3.9–4.8 h^−1^ (without the purifier) to 6.5–6.7 h^−1^, corresponding to a 50% reduction in indoor PM and PN after 10–15 min of switching off the aerosol source. The efficiency of the HEPA air purifier exceeded 95% in capturing ultrafine and coarse particles and ranged between 82–88% for particles in the accumulation range. This study highlighted the significance of mitigating indoor pollution in closed environments, especially in densely seated classrooms where the infection risk of viruses’ transmission is high. The findings of this study recommend the use of HEPA air purifiers in closed environments, especially when the ventilation system is not equipped with an efficient in-line filter. 

## Figures and Tables

**Table 1:** Characteristics of the investigated classrooms.

No.	Classroom	Building Name	Number of Students	Room Height (m)	Floor Area (m^2^)	Volume (m^3^)
1	OHE136	Olin Hall (OHE)	21	3.05	86.12	262.50
2	RTH 105	Tutor Hall (RTH)	7	3.05	97.73	297.89
3	SGM 226	Seeley G. Mudd Building (SGM)	8	3.05	63.64	193.97
4	GFS 221	Grace Ford Salvatori Hall (GFS)	20	3.05	36.42	111.00
5	GFS 205	Grace Ford Salvatori Hall (GFS)	12	3.05	36.51	111.29
6	KAP159	Kaprielian Hall (KAP)	20	3.05	37.63	114.68
7	OHE 120	Olin Hall (OHE)	6	3.05	56.49	172.17
8	KDC 236	Glorya Kaufman International Dance Center (KDC)	26	3.05	89.00	271.28
9	THH 118	Taper Hall (THH)	22	3.05	76.83	234.18

**Table 2:** Indoor, ambient, and indoor-to-outdoor (I/O) ratios of PM and PN in the three measurement phases. LOD refers to the limit of detection of the employed instrument.

	**PM_2.5_ Mass Concentration (PM) (µg/m^3^)**								
	**First Phase**			**Second Phase**			**Third Phase**		
	**Indoor**	**Outdoor**	**I/O**	**Indoor**	**Outdoor**	**I/O**	**Indoor**	**Outdoor**	**I/O**
Classroom 1	1.19	7.90	0.15	0.07	2.04	0.03	0.21	9.00	0.02
Classroom 2	0.31	8.2	0.04	0.48	46.01	0.01	<LOD	24.33	NA
Classroom 3	8.62	19.55	0.44	1.25	3.04	0.41	10.97	42.39	0.26
Classroom 4	1.29	10.60	0.12	0.61	9.56	0.06	2.03	28.22	0.07
Classroom 5	1.24	13.44	0.09	1.04	10.31	0.10	0.73	6.60	0.11
Classroom 6	1.00	5.63	0.18	0.15	2.75	0.05	0.77	22.93	0.03
Classroom 7	0.27	7.83	0.03	<LOD	4.08	NA	<LOD	11.67	NA
Classroom 8	0.95	5.69	0.17	2.99	33.91	0.09	2.20	60.25	0.04
Classroom 9	2.43	21.67	0.11	1.92	18.71	0.10	0.87	11.85	0.07
	**Particle Number Concentration (PN) (particles/cm^3^)**								
	**First Phase**			**Second Phase**			**Third Phase**		
	**Indoor**	**Outdoor**	**I/O**	**Indoor**	**Outdoor**	**I/O**	**Indoor**	**Outdoor**	**I/O**
Classroom 1	207.4	8523.5	0.02	90.6	4798.5	0.02	84.0	2972.8	0.03
Classroom 2	113.73	2290.07	0.05	165.87	5285.31	0.03	42.76	3672.92	0.01
Classroom 3	2328.7	6693.6	0.35	1800.0	5557.3	0.32	1917.8	7050.2	0.27
Classroom 4	258.6	5537.9	0.05	306.2	6704.9	0.05	253.8	6944.3	0.04
Classroom 5	345.6	8453.6	0.04	151.0	8414.1	0.02	363.1	17704.5	0.02
Classroom 6	1389.0	14338.6	0.10	299.1	12158.4	0.02	89.4	10312.7	0.01
Classroom 7	76.36	6215.27	0.01	52.33	4425.42	0.01	53.41	5573.16	0.01
Classroom 8	388.0	13783.6	0.03	220.0	7050.2	0.03	90.4	5807.8	0.02
Classroom 9	1100.2	17763.5	0.06	673.5	14862.2	0.05	543.5	15294.1	0.04

**Table 3:** Theoretical versus experimental decay rates for particle mass (PM) with and without the use of purifier at different settings in classroom 3.

	Theoretical K (h^−1^)	Experimental K (h^−1^)
Without purifier (K_natural_)	4.32	4.74 ± 0.15
Purifier at low setting (K_purifier (low)_)	5.22	5.09 ± 0.13
Purifier at high setting (K_purifier (high)_)	6.90	6.70 ± 0.33

## Data Availability

The data that support the findings of this study are available on request from the corresponding author.

## Supplementary Materials

The following supporting information can be downloaded at: https://www.mdpi.com/article/10.3390/ijerph192114558/s1. Figure S1: Number-based size distribution curve of NaCl particles; Figure S2: Actual *AER* measured in the selected classrooms in comparison with the *AER* received from USC facilities and management (FM) department; Figure S3: PM and PN measurements in classroom 3 (a) without using purifier, (b) with purifier at low flow rate (267 m^3^/h), and (c) with purifier at maximum flow rate (748 m^3^/h). References [70,71] are cited in Supplementary Materials.
