# Supplementary material for: The Role of Portable Air Purifiers and Effective Ventilation in Improving Indoor Air Quality in University Classrooms
Source: Int J Environ Res Public Health. 2022 Nov 6;19(21):14558. doi: 10.3390/ijerph192114558 (PMC9658982; doi:10.3390/ijerph192114558)
Supplement: Supplementary file 1 [file ijerph-19-14558-s001.zip › ijerph-1926695-supplementary.pdf]

International Journal of
Environmental Research and Public Health

MDPI

Article

The role of portable air purifiers and effective ventilation in improving indoor air quality in university classrooms

Mohammad Aldekheel [1,2], Abdulmalik Altuwayjiri [3], Ramin Tohidi [1], Vahid Jalali Farahani [1] and Constantinos Sioutas [1,*]

[1] Department of Civil and Environmental Engineering, University of Southern California, Los Angeles, CA 90089, USA
[2] Department of Civil Engineering, Kuwait University, P.O Box 5969, Kuwait City 13060, Kuwait
[3] Department of Civil and Environmental Engineering, College of Engineering, Majmaah University, AL-Majmaah 11952, Saudi Arabia

* Correspondence: sioutas@usc.edu; Tel.: +1 213-740-6134; Fax: +1 213-744-1426

Supplementary Information

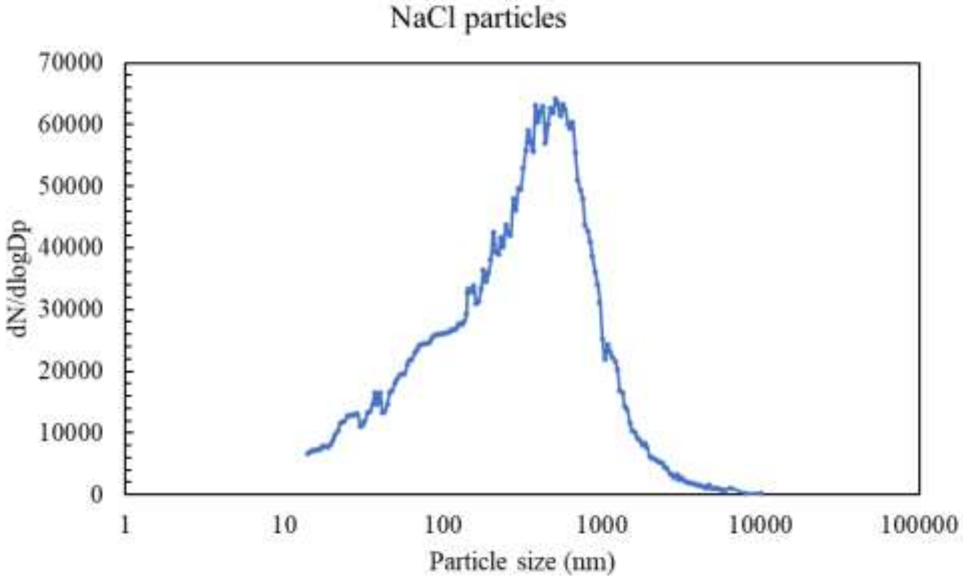

Figure S1. Number-based size distribution curve of NaCl particles

Calibration of the monitoring instruments

All our instruments were calibrated before the beginning of our monitoring campaigns. The DustTrak was calibrated by the manufacturer using A1 test dust (Arizona Test Dust) and was assigned with a calibration factor of 1. In order to assure the accuracy of the mass concentration readings for the specific aerosol used in our experiments (NaCl), we calibrated the instrument using a reference gravimetric method to obtain a new calibration factor. We used the personal cascade impactor sampler (PCIS) (Model 225-370, SKC Inc., USA) as a reference gravimetric method and equipped with a 37 mm PTFE (Pall Life Sciences Inc., USA). We aerosolized NaCl particles and performed a 2-hour sampling using both the DustTrak and the PCIS operating in parallel. Afterward, the new calibration factor was calculated using the equation below:

$$New\ calibration\ factor = \frac{Reference\ mass\ concentration}{DustTrak\ mass\ concentration} x Old\ calibration\ factor \tag{S1}$$

The old calibration factor was equal to 1 for A1 test dust. The new calibration factor was calculated for the NaCl test aerosol using the above equation and found to be 0.89. The OPS was calibrated by the manufacturer using Polystyrene Latex (PSL) particles with a calibration factor of 1 as well. The calibration procedure of TSI OPS was the same as the TSI DustTrak by performing gravimetric analysis and obtaining a new test aerosol calibration factor. The calibration of the Q-track was also performed by the manufacturer prior to our campaign by passing pure air to perform the zero calibration and employing a cylinder of known CO_2 concentrations to conduct the span calibration.

The methodology of calculating air exchange rates inside classrooms

In this study, CO_2 was chosen as the tracer gas since it is a non-reactive gas and was a readily available source (i.e., students) in our studied classrooms. The AER was calculated in this study based on the approach in one of our earlier studies by Fruin et al. (2011) [70], in which AER values were determined inside moving vehicles instead of a classroom. After approximately 10 minutes from the beginning of each lecture, CO_2 concentration increased until reaching a well-mixed steady-state condition. CO_2 reached the equilibrium concentration when the production of CO_2 by students was equal to the losses of CO_2 due to air circulation in the ventilation system. The AER was calculated in the classrooms using the mass balance equation below:

$$\frac{dC_{in}}{dt} = \frac{S}{V} + (C_{out} - C_{in})AER - C_{in}k \tag{S2}$$

where dC_{in}/dt is the change of indoor CO_2 concentration with time, S/V is the CO_2 emission rate (ppm/hr), k is the deposition rate of CO_2 (hr^{-1}), AER is the air exchange rate (hr^{-1}), C_{in} and C_{out} are the indoor and outdoor CO_2 concentrations (ppm), respectively. CO_2 is a conservative and non-reactive gas; therefore, it will remain suspended in the air since its decay is very slow (i.e., k = 0). The CO_2 emission rate (S/V) was calculated by measuring the CO_2 build-up rate from students. Before reaching the steady-state concentration, the increase in CO_2 concentrations with time was linear and the build-up rate was determined as the slope of the line [70,71]. Once CO_2 equilibrium concentration is reached (i.e., $dC_{in}/dt = 0$), Equation (S2) can be rearranged to the following equation:

$$AER = \frac{S}{V\,(C_{in} - C_{out})} \tag{S3}$$

Equation (S3) was used to calculate the air exchange rate in the 9 analyzed classrooms. For each classroom, the AER was calculated on three different days and then compared with the AER values received from the facilities and management department.

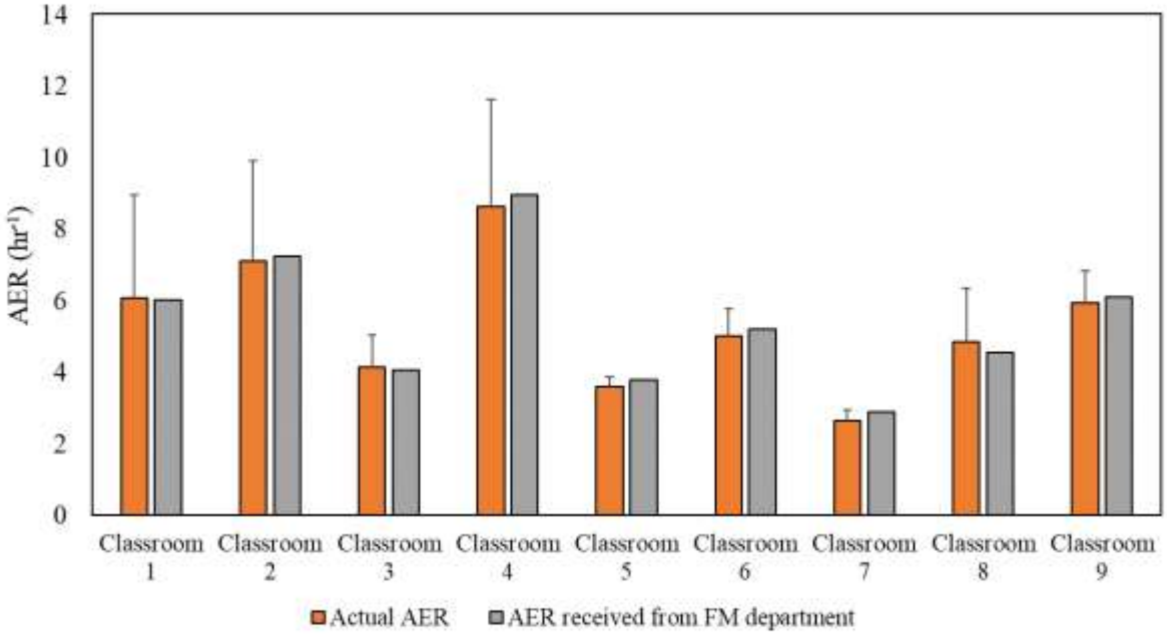

Figure S2. Actual AER measured in the selected classrooms in comparison with the AER received from USC facilities and management (FM) department.

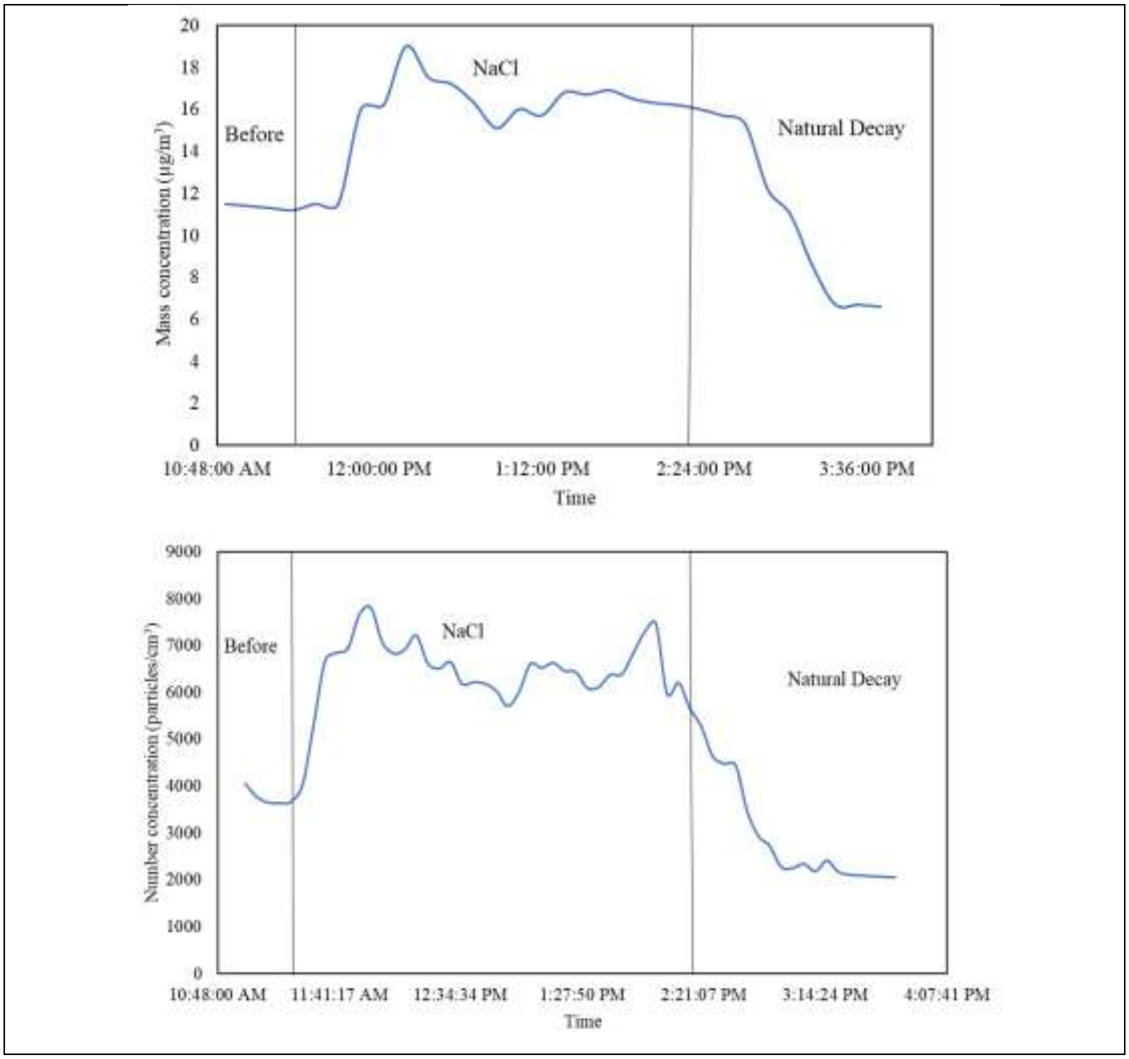
Mass concentration (µg/m³)
Before
NaCl
Natural Decay
Time
10:48:00 AM
12:00:00 PM
1:12:00 PM
2:24:00 PM
3:36:00 PM
Number concentration (particles/cm³)
Before
NaCl
Natural Decay
Time
10:48:00 AM
11:41:17 AM
12:34:34 PM
1:27:50 PM
2:21:07 PM
3:14:24 PM
4:07:41 PM

(a)

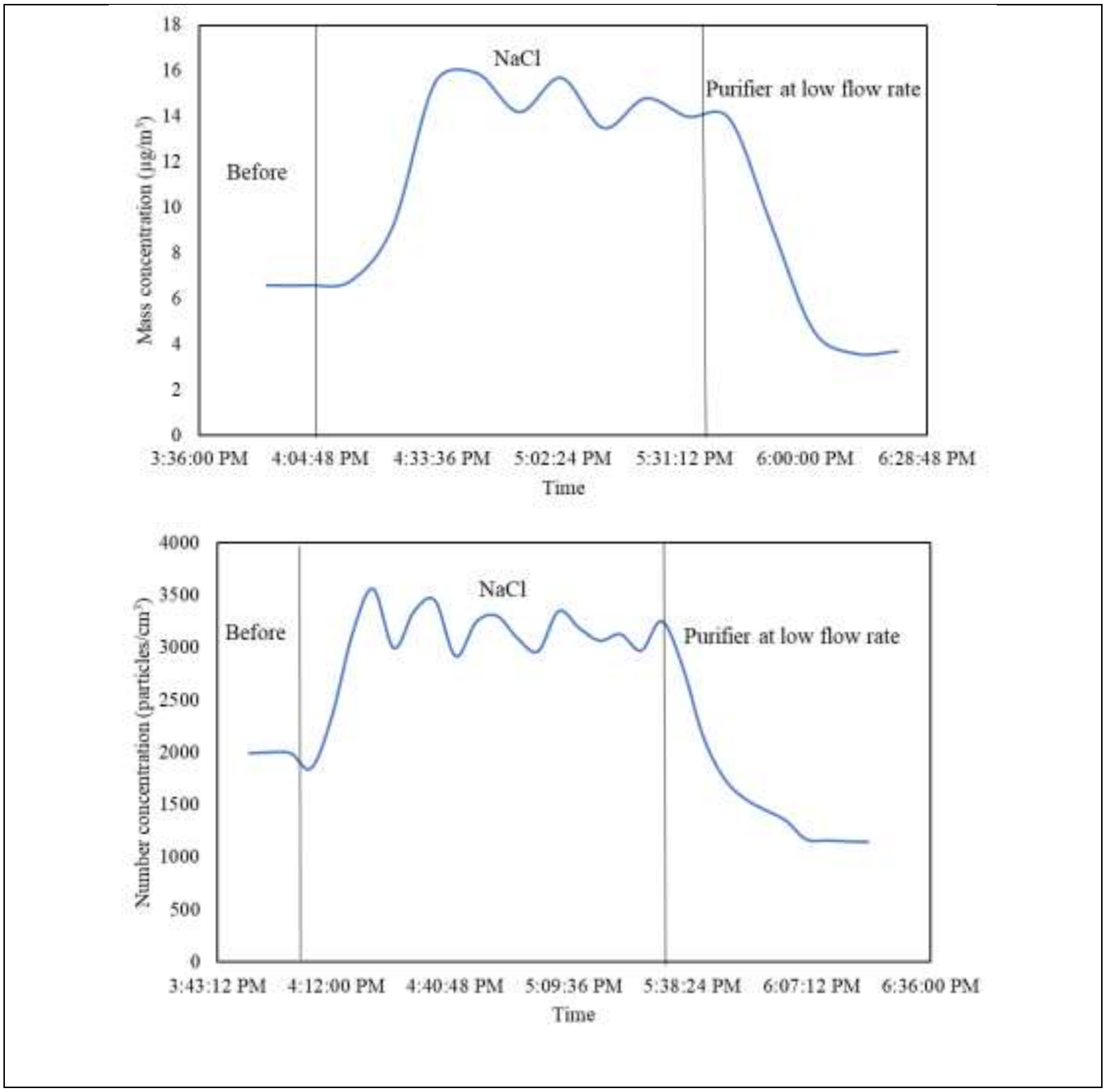
18
16
14
12
10
8
6
4
2
0
Mass concentration (μg/m³)
NaCl
Purifier at low flow rate
Before
3:36:00 PM
4:04:48 PM
4:33:36 PM
5:02:24 PM
5:31:12 PM
6:00:00 PM
6:28:48 PM
Time
4000
3500
3000
2500
2000
1500
1000
500
0
Number concentration (particles/cm³)
NaCl
Before
Purifier at low flow rate
3:43:12 PM
4:12:00 PM
4:40:48 PM
5:09:36 PM
5:38:24 PM
6:07:12 PM
6:36:00 PM
Time

(b)

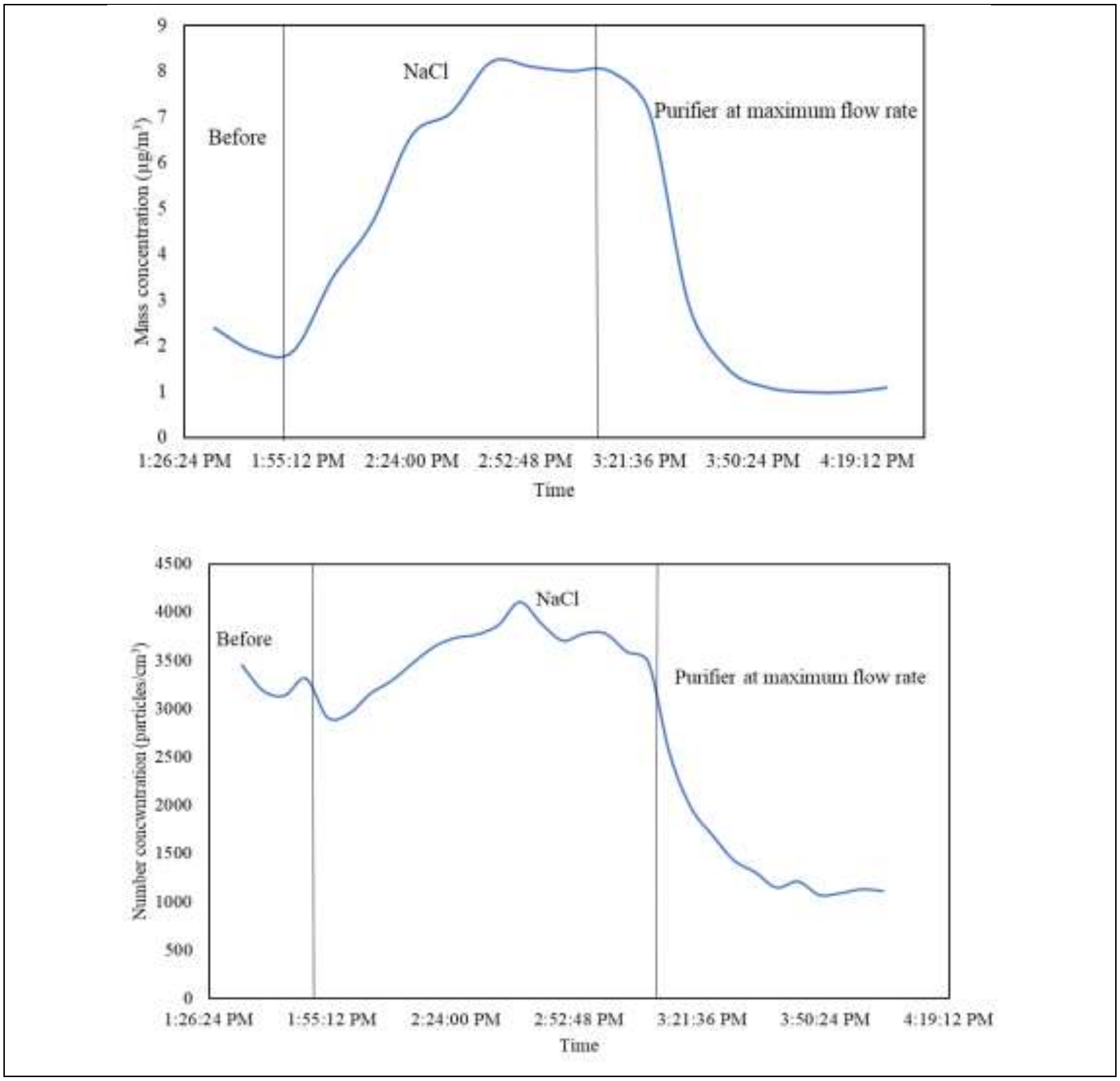

(c)

Figure S3. PM and PN measurements in classroom 3 (a) without using purifier, (b) with purifier at low flow rate (267 m^3/h), and (c) with purifier at maximum flow rate (748 m^3/h)
